# Supplementary figures and images for: Genome-wide CRISPR screenings identified SMCHD1 as a host-restricting factor for AAV transduction
Source: PLoS Pathog. 2024 Jul 8;20(7):e1012344. doi: 10.1371/journal.ppat.1012344 (PMC11257396; doi:10.1371/journal.ppat.1012344)

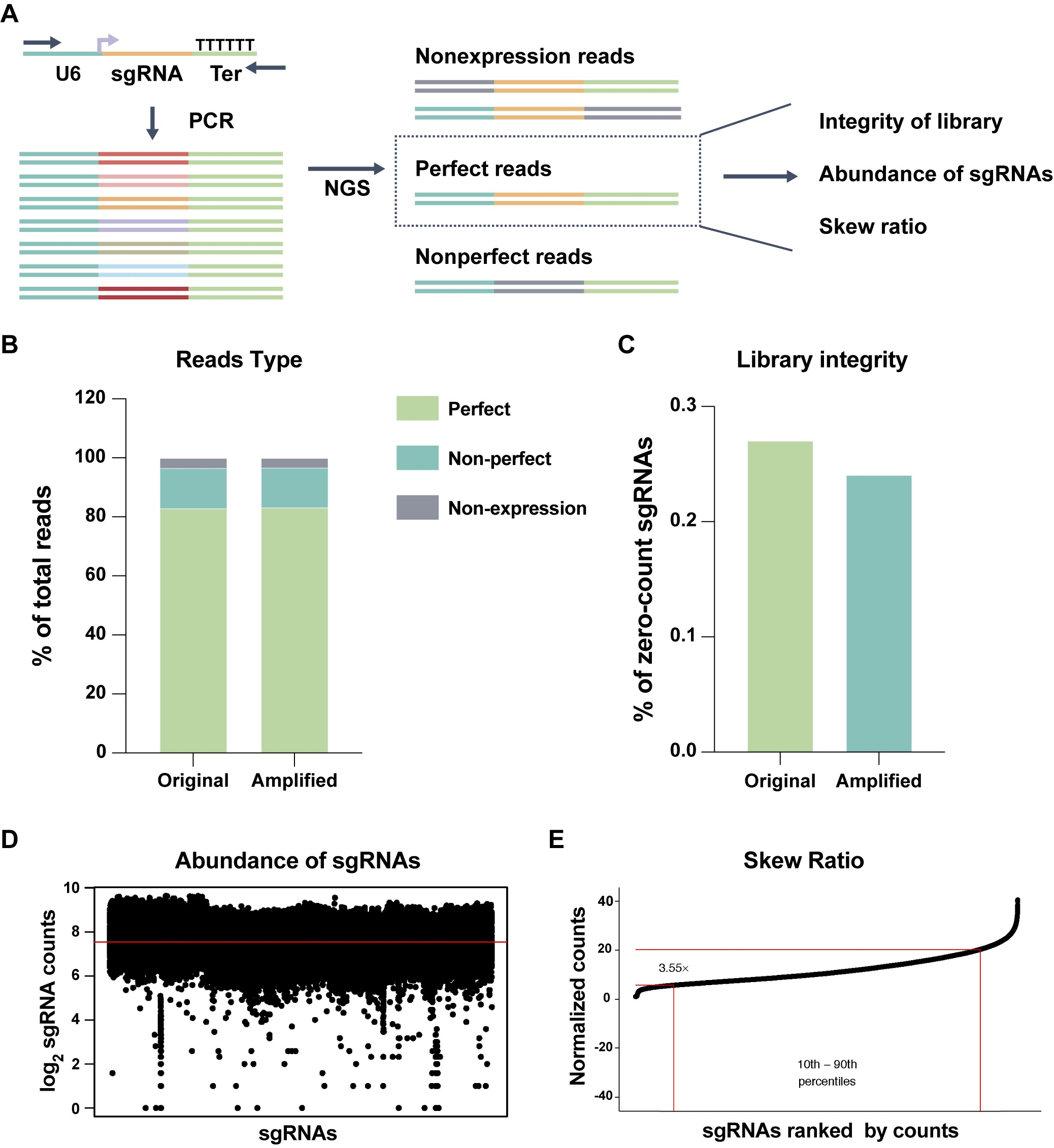

Supplement: S1 Fig — (A) Schematic of quality evaluation for the amplified CRIPSR library. Brunello library was amplified according to the Addgene protocol (cat. 73178). Amplicon comprising U6 region, sgRNAs, terminator (TTTTT) was prepared for NGS. Total sequencing reads were defined as (1) Non-expression reads, which there were mismatches in U6 or terminator region; (2) Non-perfect reads, which there were mismatches in sgRNA region; (3) Perfect reads, which were perfectly aligned with expected sequences. Counts of perfect reads were used to evaluate library integrity, abundance of sgRNAs, and skew ratio of sgRNA abundance. (B) Quality control of NGS data. The majority of sequencing reads were perfectly aligned in both NGS data. (C) Amplified library maintained most of the original sgRNAs. (D, E) Distribution of sgRNAs was uniform in the amplified library. The red line in (D) indicated the median counts of sgRNAs. Skew ratio was calculated by dividing the 90th percentile by the 10th percentile normalized sgRNA counts. (TIF) [file ppat.1012344.s001.tif]

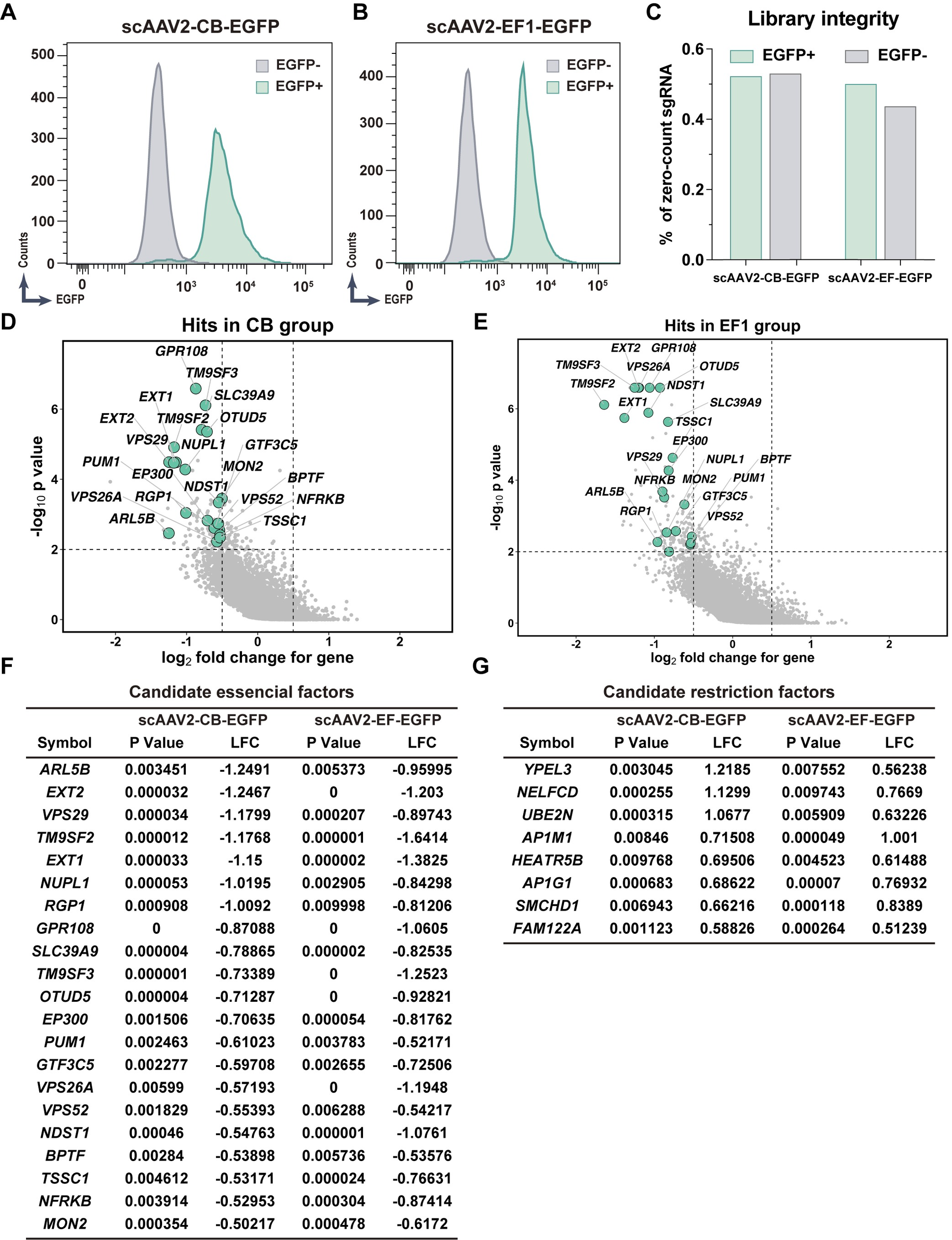

Supplement: S2 Fig — (A, B) EGFP+ and EGFP- populations were sorted from scAAV2 transduced cells. (C) Entire library was delivered into cells. Zero-count sgRNAs in EGFP+ and EGFP- cells were less than 1%. (D, E) Candidate essential factors of AAV transduction. Candidates were defined as the hits only if outstanding in both scAAV2-CB-EGFP and scAAV2-EF1-EGFP screenings. Data were analyzed by MAGeCK, and the EGFP- group was defined as the control. Hits were defined according to the following indicators calculated by MAGeCK: (1) p < 0.01, (2) LFC < -0.5, and (3) number of good sgRNA > 1. (F, G) Information of candidates in both scAAV2-CB-EGFP and scAAV2-EF1-EGFP screenings. LFC, logarithm of fold change with base 2. (TIF) [file ppat.1012344.s002.tif]

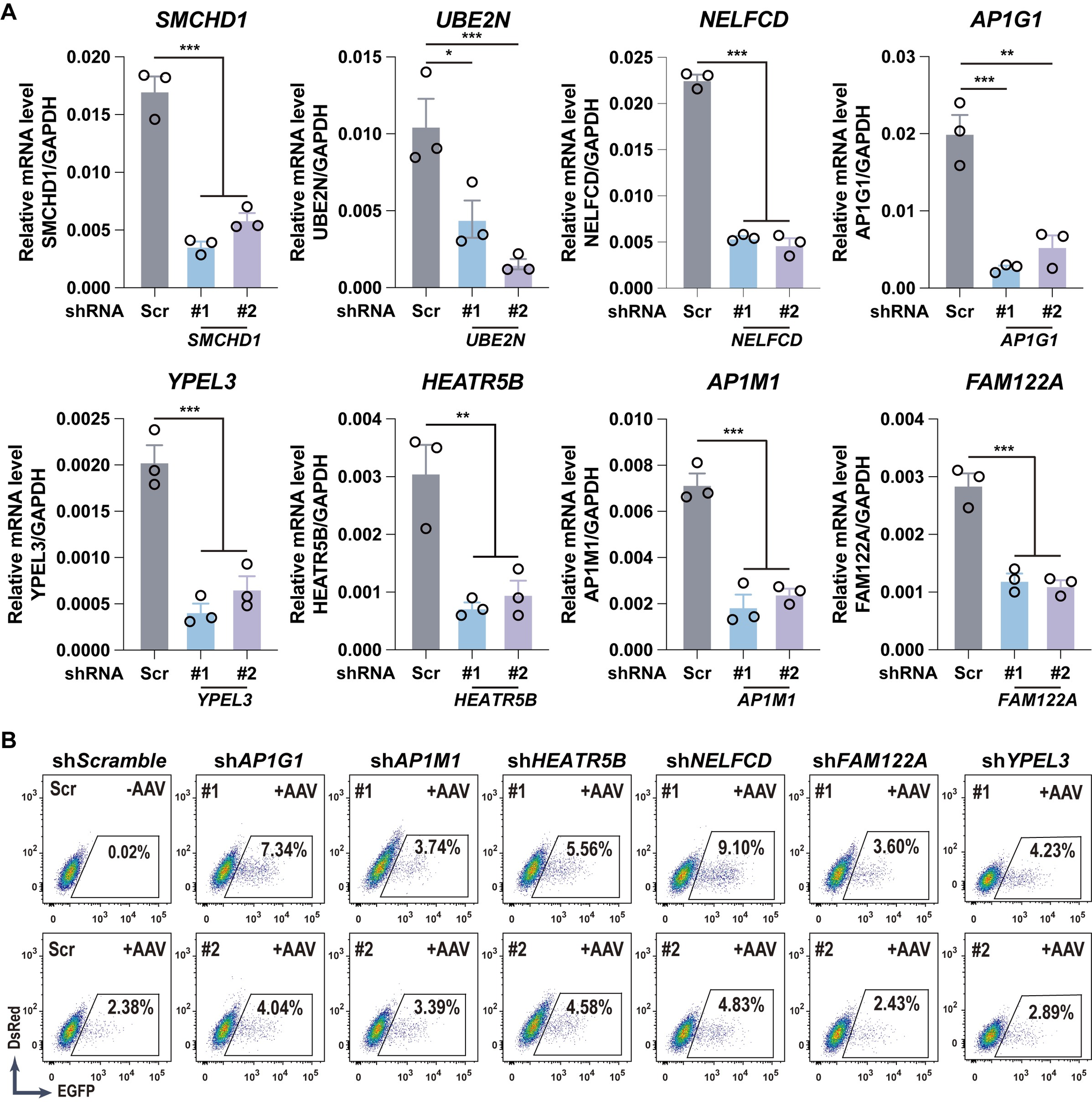

Supplement: S3 Fig — (A) RT-qPCR confirmed knockdown of candidate in HeLa. Error bar represented data from three independent experiments. Statistics: One-way ANOVA by SPSS v29.0. ***p < 0.001, **p < 0.01, *p<0.05. (B) Percentage of EGFP+ cells were quantified by FACS. All the eight candidates were separated into three groups for validation. AP1G1, AP1M1, and HEATR5B for group 1. SMCHD1, UBE2N, and YPEL3 for group 2. NELFCD and FAM122A for group 3. Representative data from FACS were shown. (TIF) [file ppat.1012344.s003.tif]

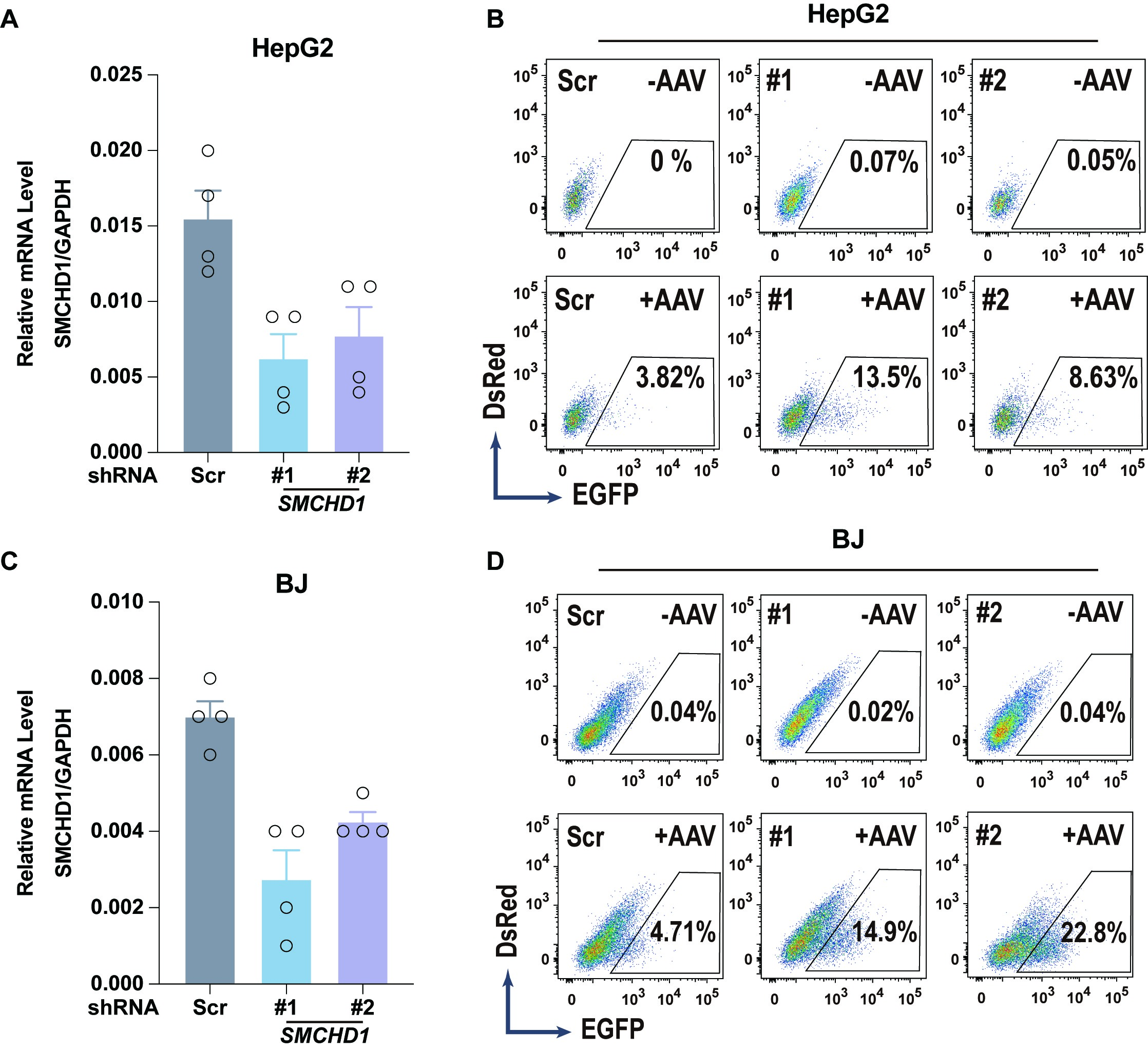

Supplement: S4 Fig — (A) RT-qPCR confirmed knockdown of SMCHD1 in HepG2. Error bar represented data from two independent experiments. (B) SMCHD1-KD significantly enhanced scAAV2-CB-EGFP transduction in HepG2 cells. (C) RT-qPCR confirmed knockdown of SMCHD1 in BJ. Error bar represented data from two independent experiments. (D) SMCHD1-KD significantly enhanced scAAV2-CB-EGFP transduction in BJ cells. (TIF) [file ppat.1012344.s004.tif]

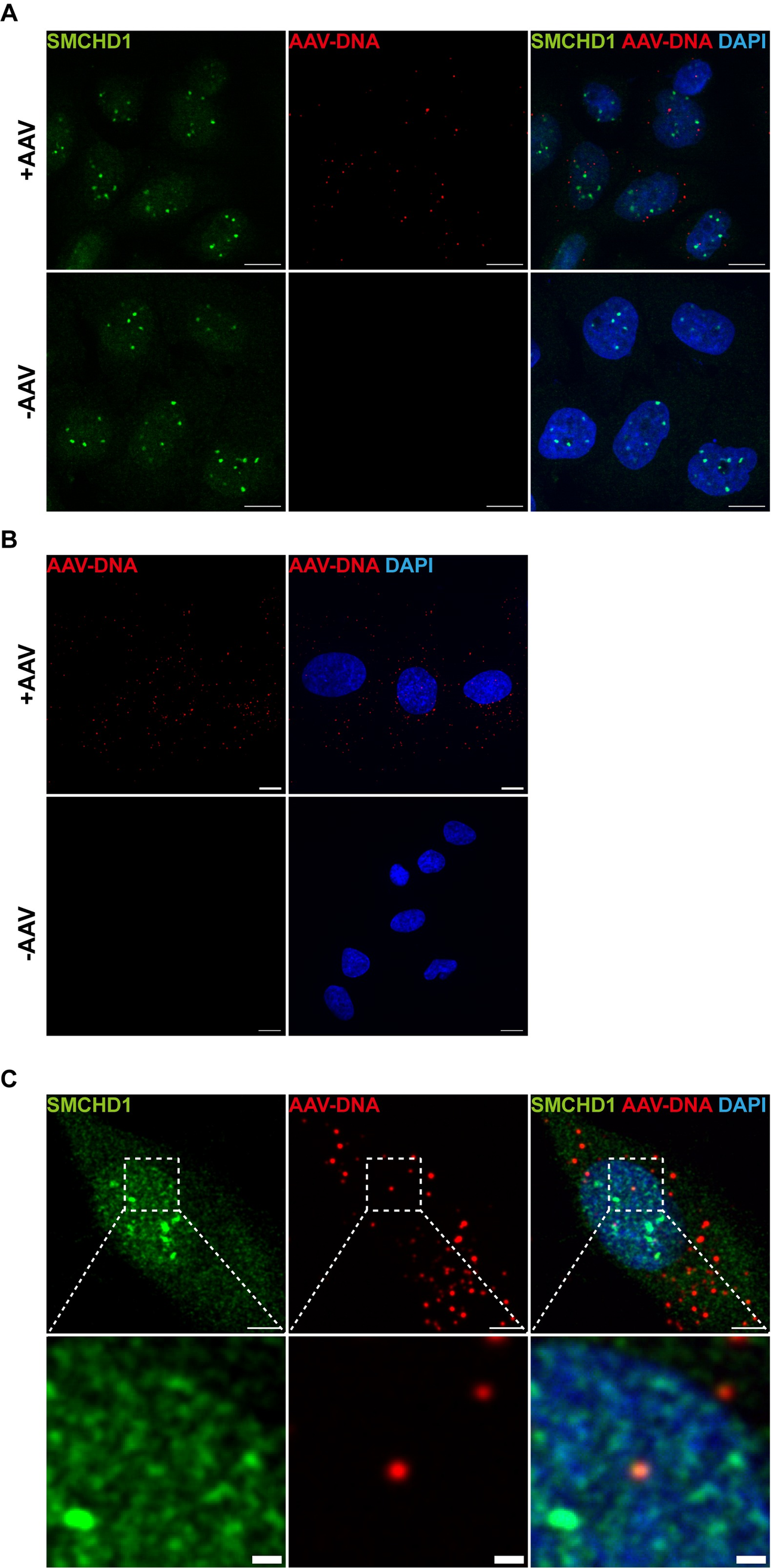

Supplement: S5 Fig — (A) Probe for luciferase was specific to vDNAs in HeLa cells. Both transduced and untransduced HeLa cells were fixed and immunostaining with anti-SMCHD1 antibody and luciferase DNA-targeting probe. Scale bars: 10μm. (B) Probe for luciferase was specific to vDNAs in BJ cells. Scale bars: 10μm. (C) Co-localization of endogenous SMCHD1 with AAV DNAs in BJ cells. Scale bars: 5μm and 1μm (zoom-in). (TIF) [file ppat.1012344.s005.tif]

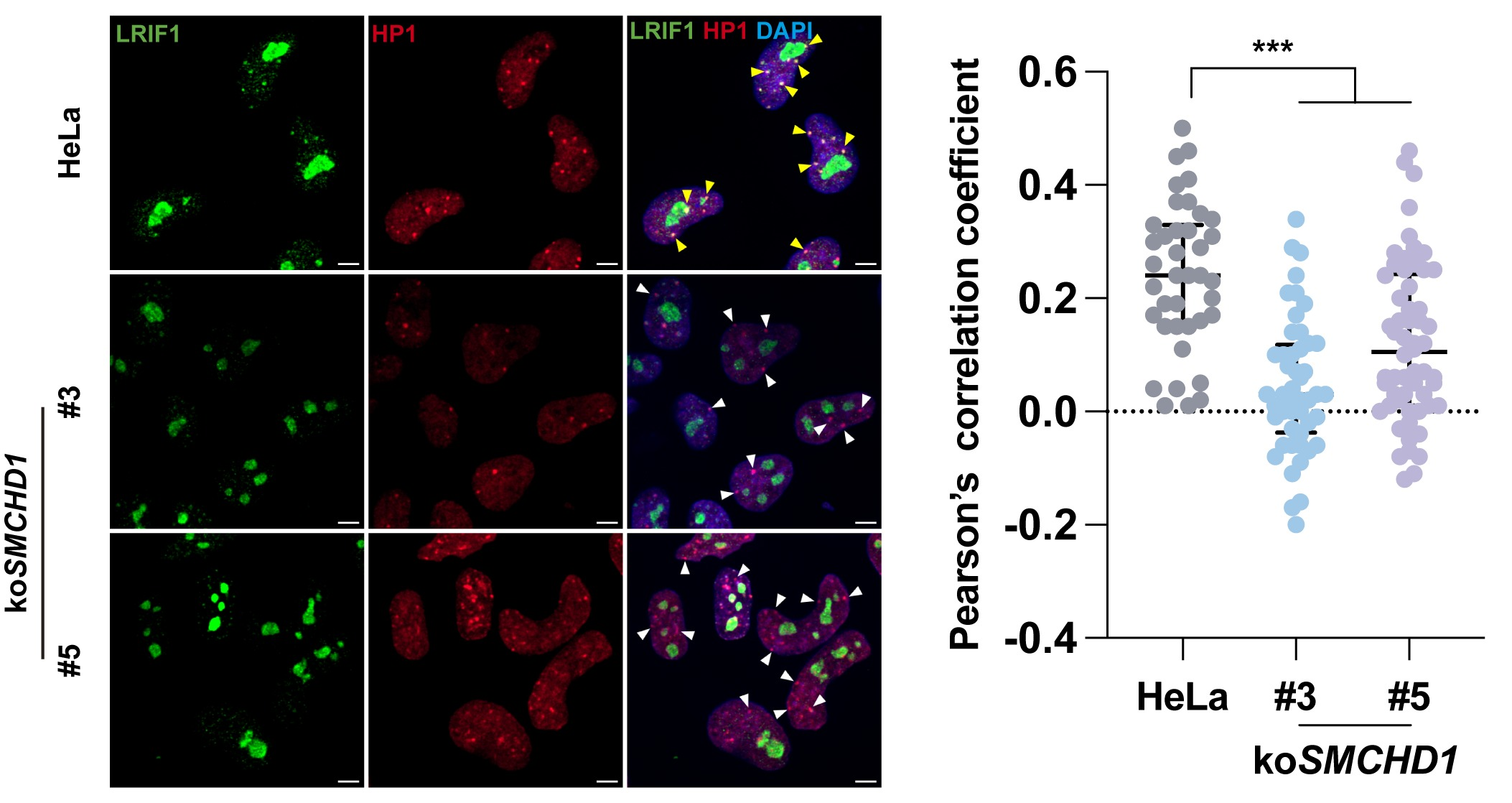

Supplement: S6 Fig — Co-localization of LRIF1 and HP1 were detected by IF. Yellow arrowheads, co-localization of LRIF1 and HP1. White arrowheads, disrupted co-localization of LRIF1 and HP1. Scale bar: 5μm. Co-localization was quantified using PCC methods by coloc2 plugin in Image J. Each dot represents a single cell. Statistics: One-way ANOVA by SPSS v29.0. ***p<0.001. (TIF) [file ppat.1012344.s006.tif]

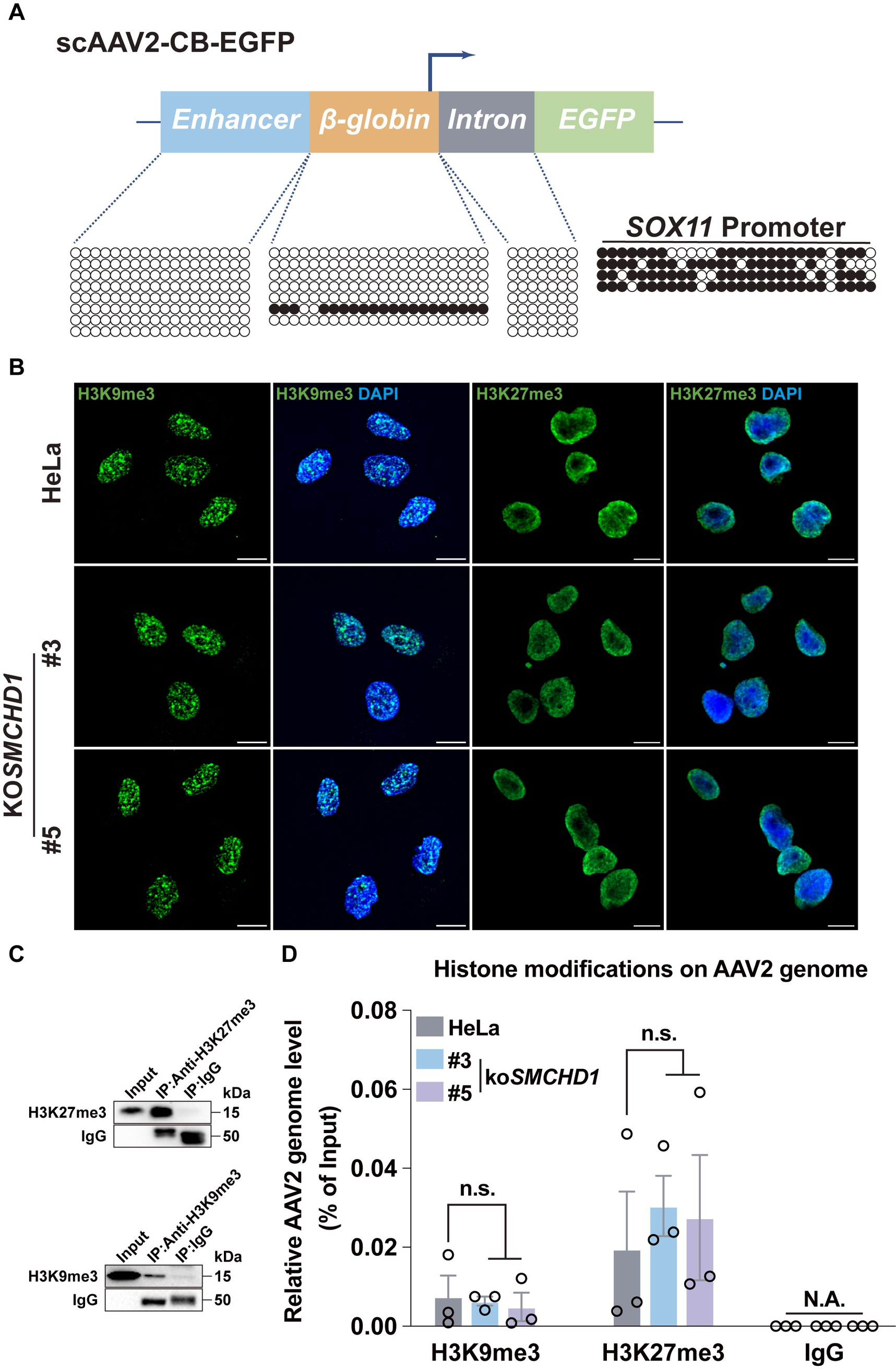

Supplement: S7 Fig — (A) Lack of intense DNA methylation in AAV genomic regions. The transcriptional regulating elements in the scAAV2-CB-EGFP genome included a CMV enhancer, a chicken beta-globin promoter, and a SV40 intron from 5’ to 3’. HeLa cells were transduced with scAAV2-CB-EGFP (MOI: 20000 vgs/cell), and the genomic DNA was extracted for bisulfate conversion. Amplicons covering the regulating elements were cloned for sanger sequence. Each circle indicates a CpG dinucleotide. Solid circle means the methylated CpG site while hollow circle indicates the unmethylated CpG dinucleotide. SOX11 promoter sequence was used as a positive control for CpG methylation. (B) SMCHD1-KO did not alter the overall H3K9me3 and H3K37me3 modifications in nucleus. (Scale bar: 10μm). (C) Immunoprecipitation of H3K9me3 and H3K27me3. Hela cells were lysed using RIPA buffer and protein samples were used for immunoprecipitating. Normal IgG worked as control. (D) SMCHD1-KO did not affect H3K9me3 and H3K37me3 modifications on AAV genome. HeLa or SMCHD1-KO cell lysate (NP-40 lysis buffer) transduced with scAAV2-CB-EGFP (MOI: 1000 vgs/cell) were used to test the levels of H3K9me3 and H3K37me3 modifications on AAV genome by immunoprecipatitation with anti-H3K9me3 and anti-H3K37me3 antibodies. Normal IgG was used as control. Level of co-immunoprecipitated AAV genome DNA was quantified by qPCR. Error bar represented data from three independent experiments. Statistics: One-way ANOVA by SPSS v29.0, n.s., not significant. (TIF) [file ppat.1012344.s007.tif]
