# Supplementary material for: Genome-wide CRISPR screenings identified SMCHD1 as a host-restricting factor for AAV transduction
Source: PLoS Pathog. 2024 Jul 8;20(7):e1012344. doi: 10.1371/journal.ppat.1012344 (PMC11257396; doi:10.1371/journal.ppat.1012344)
Supplement: Attachment — Submitted filename: 20240604-Responses to reviewers comments-LZH.pdf [file ppat.1012344.s014.pdf]

Reviewer #1:

In this manuscript, Wang et al. use genome-wide CRISPR screening to identify host factors that restrict AAV transgene expression. As low transgene expression is thought to be a significant contributor to the high viral loads needed for AAV in gene therapy applications, an improved understanding of the mechanisms regulating AAV transgene expression is important for devising potential strategies to improve therapeutic approaches.

In general, the manuscript is clearly written and the data, for the most part, are well controlled, robust, and compelling. The identification and validation of SMCHD1 as a restriction factor for AAV gene expression is especially thorough, using multiple ways of targeting SMCHD1 and multiple cell types and AAV serotypes. The authors go on to characterize the mechanism by which SMCHD1 represses AAV transgene expression, concluding it does so through a complex with LRIF1 and HP1, likely resulting in condensation and repression of viral chromatin. Unfortunately, the authors do not directly test key aspects of their mechanistic model, namely that SMCHD1 and LRIF1 repress AAV gene expression via HP1 and chromatin condensation, leaving the mechanistic aspect of the manuscript somewhat incomplete.

Major comments：

(1) The authors should test HP1 association with viral DNA in SMCHD1 and/or LRIF1 deficient cells. Their model suggests that SMCHD1/LRIF1 suppress AAV2 gene expression via HP1 and chromatin condensation, but they do not present experiments that directly test this.

Response:

Thanks for the critical comment. In the revised manuscript, we first tested the change of AAV genome accessibility in wt, *SMCHD1*-KO and *LRIF1*-KD HeLa cells by ATAC-qPCR, which would be an indicator of chromatin state. The AAV genome accessibility was indeed strongly increased upon *SMCHD1* knockout, while it did not affect actively transcribed genes such as *ACTB* and *TUBB3* nor the desert locus (**Fig. 6g**). For *LRIF1*-KD cells, a modest but significant increase of AAV genome accessibility was also detected (**Fig. 6h**), which might be affected by the residual LRIF1 still present in the cells. Notably, neither *SMCHD1*-KO nor *LRIF1*-KD would affect HP1A protein level (**Fig. 5a, i**) and its association with AAV genome as indicated by immunoprecipitation of HP1A and qPCR analysis of bound AAV genomic DNAs (**Fig. 6a-b, d-e**). Together, these data supported that interruption of the SMCHD1/LRIF1 would increase AAV genome accessibility, leading to increased gene transcription, while not affecting HP1A expression and its viral genome interaction.

(2) The data shown in Fig. 5 show that HP1 is associated with viral genomes in wild-type cells, but do not test whether that association is abrogated or diminished in the absence of SMCHD1 and/or LRIF1.

Response:

Thanks for the comment. As mentioned above, in the revised manuscript, we did immunoprecipitation of HP1A in *SMCHD1*-KO and *LRIF1*-KD HeLa cells and quantified HP1A-bound AAV DNAs by qPCR. The results indicated that neither *SMCHD1*-KO nor *LRIF1*-KD would affect HP1A protein level and its association with the AAV genome (**Fig. 6a-c, d-f**).

(3) Similarly, are the HP1 co-localization experiments shown in Fig. 5 and Fig. S8 performed in transduced or untransduced cells? Can the authors show that HP1 co-localizes with viral DNA and, if so, whether loss of SMCHD1 and/or LRIF1 disrupts that localization?

Response:

Thanks for the comment. The original co-localization experiments were done in untransduced cells. In the revised manuscript, we have provided representative co-localization images for HP1 with AAV genomic DNAs in transduced HeLa cells (**Fig. 5h**). We also did quantification of HP1α binding with AAV genomic DNAs upon *SMCHD1*-KO and *LRIF1*-KD by qPCR and imaging analysis, both of which indicated that HP1α's association with viral DNAs were not significantly changed (**Fig. 6b-c, e-**

f). These data suggested that SMCHD1-LRIF1 complex only regulated chromatin state of AAV genomes but not its existing association with HP1 proteins.

(4) Is HP1 binding required for repression by SMCHD1 / LRIF1? Can the authors show whether reconstitution of LRIF1 KD cells with a HP1 or SMCHD1 binding mutant (ref. 43) fails to rescue repression of AAV gene expression? Such a result would strengthen the authors' mechanistic conclusions.

Response:

Thanks for the critical comment. In the revised manuscript, we constructed two *LRIF1* mutants, one without SMCHD1-binding domain (m1) and the other with neither HP1 binding motif nor SMCHD1-binding domain (m2) (**Fig. 5b**). Immunoprecipitation confirmed that m1 indeed lost the interaction with SMCHD1 while m2 lost both HP1 and SMCHD1 association (**Fig. 5c**). AAV transduction results indicated that neither m1 nor m2 would rescue the repression of AAV gene expression in *LRIF1*-KD cells (**Fig. 5d-e**). These results suggested that LRIF1's association with both SMCHD1 and HP1 was required to repress AAV gene expression.

Minor comments：

(1) Does repopulation of SMCHD1 KO cells with SMCHD1 reverse the

enhancement of AAV2 gene expression? Similar to what the authors have shown for repopulation with LRIF1 in Fig. 4g? The authors have robustly shown SMCHD1 deficiency results in increased AAV gene expression, but demonstrating they can reverse that by re-introduction of SMCHD1 would further bolster this conclusion.

Response:

Thanks for the comment. In the revised manuscript, we have conducted the rescue experiment with FLAG-SMCHD1 in wt and *SMCHD1*-KO cells transduced with luciferase expressing AAVs (**Fig. 2g-h**). The results supported that re-introduction of SMCHD1 would restore the suppression of AAV gene expression.

(2) Fig 3e: the authors should include an untransduced control to demonstrate the specificity of their AAV DNAscope. It would also be helpful to quantitate this and other microscopy data such as Fig. 4a, 5a, and 5d to show the degree to which colocalization does or does not occur across the population of cells.

Response:

Thanks for the comment. In the revised manuscript, we have included the untransduced Hela control data and DNAscope signals were only observed in the transduced cells (**Fig. S5a**). All the above mentioned figures were also updated with quantitative imaging analysis (**Fig 4a, 5a, i and S6**),

which measures Pearson's coefficients by coloc2 plugin in Image J [1] and is used in several other recent imaging studies [2, 3].

(3) Is repression by SMCHD1 dependent on LRIF1? The authors demonstrate in Fig. 4 that repression by LRIF1 is dependent on SMCHD1, but because the data in Fig 4f appears to be normalized to the Scr condition for each cell line, it is impossible to evaluate whether enhancement of AAV2 gene expression by SMCHD1 knockout is dependent on LRIF1.

Response:

Thanks for the comment. In the revised manuscript, we have adjusted the way of data presentation by normalizing all the data to scramble control in wt Hela cells (**Fig. 4g**). *SMCHD1*-KO resulted in increased AAV transduction, while the effect of *LRIF1*-KD was largely diminished in *SMCHD1*-KO cells.

(4) Fig 2. Panels a-e (SMCHD1 KO and KD in additional cell types) seem like they could better fit in Fig. 1 establishing SMCHD1 as an inhibitor of AAV2 transgene expression vs. in Fig 2 which is testing multiple serotypes of AAV.

Response:

We appreciate the reviewer's suggestion. The original Fig. 1 aimed to show representative hits identified from the genome-wide screen, while Fig. 2

was to establish SMCHD1 as a general inhibitor of AAV transduction. In the revised manuscript, due to inclusion of more data, we combined all the SMCHD1 CRISPRi and KO data into Figure 2 (**Fig. 2a-f**), as well as the newly added rescue data (**Fig. 2g-h**), in order to make the data presentation more straightforward.

(5) Figure legend for panel 3a references a western blot which is not present. I think this may actually be Fig. S7?

Response:

Thanks for pointing this out. Indeed, the western blot image referred to Figure S7. We have corrected it in the revised manuscript (**Fig. 3b**).

(6) Figure legend for Fig. 4: panels e and f appear to be swapped.

Response:

Corrected. Thanks.

Reviewer #2:

In this manuscript, Wang et al perform a genome wide CRISPR screen to determine host factors that limit AAV transduction efficiency. Among other factors, they discover that SMCHD1 restricts AAV gene expression after nuclear entry in multiple cell types and with multiple serotypes of AAV. Overall, these findings are rigorously explored using multiple techniques.

However, in multiple instances certain conclusions are over-interpreted or at least could be supported with alternative methodologies or additional quantification as detailed below.

Major comments:

(1) In Figure 3 a direct link is claimed between SMCHD1 and AAV genomes. However the standard PCR performed in Figure 3C is not quantitative or quantitated and is lacking information about the percentages of material in either the input or the IP.

Response:

Thanks for the comment. In the revised manuscript, a quantitative analysis of SMCHD1-AAV interactions with %input was included (**Fig. 3e**).

(2) Furthermore, one field of microscopy data is shown highlighting a single overlap of AAV genome and SMCHD1. This key experiment needs to be quantified over multiple fields and multiple replicates. Ideally the same experiment would also be performed on uninfected cells to highlight any potential changes in SMCHD1 morphology as well as the cleanliness of highly-amplified DNAscope experiments.

Response:

Thanks for the critical comment. In the revised manuscript, we have included quantitative analysis for SMCHD1-AAV co-localization. First,

DNAscope and anti-SMCHD1 immunostaining results in both AAV-transduced and non-transduced cells were updated in **Fig. S5a**, which indicated the DNAscope signals were specific. Next, we quantified the percentage of cells with co-localization signals in multiple fields and replicates (two independent experiments with six view fields each) and the results were updated in **Fig. 3g**.

(3) In Figure 4A need quantification over multiple fields and multiple replicates for interaction between SMCHD1 and LRIF1. Alternatively, biochemical interaction could be shown with co-immunoprecipitation.

Response:

Thanks for the comment. Quantitative analysis of co-localization between SMCHD1 and LRIF1 was done by using the Pearson's correlation coefficient (PCC) method in the Coloc2 plugin of ImageJ [1], which was also adopted in several other recent studies [2, 3]. Individual nuclear was identified according to the DAPI signals. The data has been updated in the revised manuscript (**Fig. 4a**). Co-immunoprecipitation using FLAG-tagged LRIF1, and its mutants further confirmed the interaction between SMCHD1 and LRIF1, and the interaction was lost once the binding domain was disrupted (m1) (**Fig. 5b-c**).

(4) In Figure 5A again a physical interaction is implied between both SMCHD1, LRIF1, and HP1 via one field of view of microscopy. Especially in Figure 5D where loss of LRIF1 is claimed to stop interaction of SMCHD1 with HP1 this should be validated with co-immunoprecipitation.

Response:

Thanks for the critical comment. Our initial co-immunoprecipitation experiment using anti-HP1A in Hela cells could not detect clear signal from SMCHD1, probably because only a small fraction of HP1A were bound with SMCHD1 which made it difficult to detect using western blotting. Therefore, we expressed exogenous HA-HP1A and FLAG-LRIF1 and then used the anti-HA antibody to pull down endogenous SMCHD1. The results indicated that only when LRIF1 was present, HA-HP1A could pull down endogenous SMCHD1, supporting that the interaction of SMCHD1 and HP1A was dependent on LRIF1 (**Fig. 5j**).

(5) The authors final model claims that SMCHD1-HP1 is condensing AAV genomes into an inactive state, however no experimentation has been done to support this hypothesis. It is fine to speculate about this but should be saved for the discussion.

Response:

Thanks for the comment. In the revised manuscript, we test the accessibility of AAV genome by ATAC-qPCR. Comparing *SMCHD1*-KO

with wt cells, *SMCHD1*-KO significantly increased the genome accessibility of AAV DNAs (**Fig. 6g**). As controls, no changes were detected at active transcribed and desert genomic loci (**Fig. 6g**). In addition, increased genome accessibility was also detected in *LRIF1*-KD cells (**Fig. 6h**), although to a lesser extent possibly due the presence of residue LRIF1. These data supported that the SMCHD1/LRIF1/HP1A complex might cause a suppressive state for AAV genome.

Minor comments：

(1) Panel labeling in Figure 4 is off compared to the text.

Response:

Corrected. Thanks.

(2) Line 271 is confusing (experiment using FLAG-tagged LRIF1 was carried out and supplementing the shRNA-resistant LRIF1 would abolish the AAV transcription enhancement in LRIF1-KD cells). Regardless, the data presented in Figure 4G shows a statistical decrease in the RLU after adding back in shRNA-resistant LRIF1 as described, but statistics are not performed to determine shLRIF1 increased RLU over scrambled.

Response:

The shRNA-resistant experiment was designed by mutating the shRNA targeted sequence in *LRIF1* gene so that its mRNA will not be bound by

shRNA-RISC complex for cleavage while keeping the protein sequence unchanged. The additional statistical analysis was included in the revised manuscript (**Fig. 4e**). Thanks.

(3) Figure 5C y-axis labeling is confusing. Why not simply use % Input as is done in S9.

Response:

We have modified the labeling to %Input in the revised manuscript (**Figure 5g**). Thanks.

(4) Overall the abundance of supplemental figures made this manuscript harder to read. In a journal with no prescribed figure limits it would make sense to bring at least some of this data into a more easily accessible area.

Response:

Thanks for the comment. We apologize for the inconvenience and have rearranged the figures to be more straightforward. The original Figure S3, S4c, S5, and S7 have been moved main figures (**Fig. 1c, 1f-i, 2a, and 3b**).

Reviewer #3:

In this manuscript, the authors performed a genome-wide CRISPR screen to identify factors that restrict adeno-associated virus (AAV) transduction. They identified the epigenetic modifier SMCHD1 (among others) as

crucial protein that impairs AAV gene transduction. They could demonstrate that SMCHD1 does not affect AAV attachment, entry or delivery of the genome to the nucleus, but restricts transgene expression. In addition, SMCHD1 directly binds the AAV genome and forms as complex with LRIF1 and HP1, leading to the heterochromatinization of the AAV genome. Overall, this is a well written manuscript and could contribute to the improvement in AAV gene delivery. However, a few points should be addressed prior to publication.

Major comments：

The authors show the colocalizations between the AAV genome, SMCHD1 and other proteins, but it remains elusive in what percentage of cells this occurs. Does the AAV genome and SMCHD1 colocalize in 100 % of these cells (Fig 3d)? If yes, state how many cells were analyzed. If not then properly quantify the colocalization. The same is true for the colocalization of SMCHD1, LRIF1 and HP1. Does the AAV genome and SMCHD1 also colocalize in other cell types?

Response:

Thanks for the critical comment. In the revised manuscript, we have included the quantitative imaging analysis. Specifically, for AAV DNA and SMCHD1 co-localization, we analyzed 12 random view fields from two independent experiments with a total number of 618 cells, and ~20%

cells had at least one AAV DNA spot colocalized with SMCHD1 (**Figure 3g**). The co-localization analysis of SMCHD1 with LRIF1 and HP1, we used the Pearson correlation coefficient method [1-3] and the results were updated in **Figure 4a** (SMCHD1 vs LRIF1) and **Figure 5a** (SMCHD1 vs HP1 and LRIF1 vs HP1). The co-localization of SMCHD1 with AAV DNAs was also confirmed in primary human fibroblasts (BJ cells) (**Figure S5b-c**).

Minor comments：

(1) Figure 4: the labels of the figure are partially mixed up. E.g. "...the enhancement was largely abolished in SMCHD1-KO cells (Fig. 4e)" but this is shown in panel 4f. The same is true for "...experiment using FLAG-tagged LRIF1... (Fig. 4f, g)". The Flag tag data is currently shown in Fig. 4e and 4g. Please correct and double check all figures.

Response:

We apologize for the errors and have corrected them in the revised manuscript. All the figures were double checked as well.

(2) The color scheme of Figure 4f and 4g is confusing. The LRIF1 shRNA #2 is shown in purple in 4f. So why is also the scrambled shRNA purple in 4g too? The controls were shown in grey in the other figures.

Response:

We have modified the color (**Fig. 4e** in the revised manuscript) to be clearer.

Thanks

(3) In Fig. 4g the legend for the symbols (circle or diamond) is missing.

Response:

Thanks for the comment. The symbols do not need to be differently labeled and thus all were changed to circles in the revised manuscript (**Fig. 4e**).

(4) In the summary figure 5g, it is not obvious at the first glance that the left half is the wild type scenario and the right is the SMCHD1 knockout one. Please address this by e.g. increase the fond size of these labels to make it obvious to all readers.

Response:

Thanks for the suggestion and we have modified the model image accordingly (**Fig. 7**). The font size was also increased.

References:

1. Herdman M, Isbilir B, von Kügelgen A, Schulze U, Wainman A, Bharat TAM. Cell cycle dependent coordination of surface layer biogenesis in Caulobacter crescentus. Nat Commun. 2024;15(1):3355. Epub 2024/04/19. doi: 10.1038/s41467-024-47529-5. PubMed PMID: 38637514; PubMed Central PMCID: PMCPMC11026435.
2. Patil A, Strom AR, Paulo JA, Collings CK, Ruff KM, Shinn MK, et al. A disordered region controls cBAF activity via condensation and partner recruitment. Cell. 2023;186(22):4936-55.e26. Epub 2023/10/04. doi: 10.1016/j.cell.2023.08.032. PubMed PMID: 37788668; PubMed Central PMCID: PMCPMC10792396.

3. Jovasevic V, Wood EM, Cicvaric A, Zhang H, Petrovic Z, Carboncino A, et al. Formation of memory assemblies through the DNA-sensing TLR9 pathway. Nature. 2024;628(8006):145-53. Epub 2024/03/28. doi: 10.1038/s41586-024-07220-7. PubMed PMID: 38538785; PubMed Central PMCID: PMCPMC10990941.
